# Best practice guidelines in managing the craniofacial aspects of skeletal dysplasia

**DOI:** 10.1186/s13023-021-01678-8

**Published:** 2021-01-14

**Authors:** Ravi Savarirayan, David E. Tunkel, Laura M. Sterni, Michael B. Bober, Tae-Joon Cho, Michael J. Goldberg, Julie Hoover-Fong, Melita Irving, Shawn E. Kamps, William G. Mackenzie, Cathleen Raggio, Samantha A. Spencer, Viviana Bompadre, Klane K. White, on behalf of the Skeletal Dysplasia Management Consortium

**Affiliations:** 1 Victorian Clinical Genetics Services, Murdoch Children’s Research Institute, University of Melbourne, Parkville, VIC 3052 Australia; 2 Department of Otolaryngology-Head and Neck Surgery, Johns Hopkins University School of Medicine, Baltimore, MD USA; 3 Eudowwod Division of Pediatric Respiratory Sciences, Johns Hopkins University School of Medicine, Baltimore, MD USA; 4 Division of Orthogenetics – Nemours/ A.I. duPont Hospital for Children, Wilmington, DE USA; 5 Division of Pediatric Orthopaedics, Seoul National University Children’s Hospital, Seoul, South Korea; 6 Department of Orthopedics and Sports Medicine, Seattle Children’s Hospital, Department of Orthopaedics and Sports Medicine, University of Washington, Seattle, WA USA; 7 McKusick-Nathans Department of Genetic Medicine, Johns Hopkins University, Baltimore, MD USA; 8 Department of Clinical Genetics Guy’s, St Thomas NHS, London, UK; 9 Department of Radiology, Seattle Children’s Hospital, University of Washington, Seattle, WA USA; 10 Department of Orthopedic Surgery – Nemours/ A.I. duPont Hospital for Children, Wilmington, DE USA; 11 Department of Orthopedic Surgery, Hospital for Special Surgery, New York, NY USA; 12 Department of Orthopedic Surgery, Boston Children’s Hospital, Boston, MA USA

**Keywords:** Skeletal dysplasia, Craniofacial, Best practice, Otolaryngology

## Abstract

**Background:**

Recognition and appropriate management of the craniofacial manifestations of patients with skeletal dysplasia are challenging, due to the rarity of these conditions, and dearth of literature to support evidence-based clinical decision making.

**Methods:**

Using the Delphi method, an international, multi-disciplinary group of individuals, with significant experience in the care of patients with skeletal dysplasia, convened to develop multi-disciplinary, best practice guidelines in the management of craniofacial aspects of these patients.

**Results:**

After a comprehensive literature review, 23 initial statements were generated and critically discussed, with subsequent development of a list of 22 best practice guidelines after a second round voting.

**Conclusions:**

The guidelines are presented and discussed to provide context and assistance for clinicians in their decision making in this important and challenging component of care for patients with skeletal dysplasia, in order standardize care and improve outcomes.

## Introduction

Skeletal dysplasia refers to a group of individually rare, but collectively common, conditions that affect the normal development, growth, and maintenance of the human skeleton [1, 2], including the craniofacial skeleton. Individuals with a skeletal dysplasia diagnosis have multiple medical needs, including those related to altered craniofacial growth and morphology, and ear, nose, throat, respiratory, and dental manifestations. The majority of patients with achondroplasia, for example, have obstructive sleep apnea due to adenotonsillar hypertrophy in the context of a very short basicranium and recessed midface. In patients with lysosomal storage conditions, progressive accumulation of substrates in the adenoids, tonsils and upper airway can cause obstruction. Obesity may exacerbate obstruction, just as in average stature children and adults.

Central sleep apnea may be found in patients with achondroplasia due to compression of the brainstem at the congenitally malformed foramen magnum, and in those with osteogenesis imperfecta who have symptomatic basilar invagination. Patients with predominant truncal shortening (e.g. spondyloepiphyseal dysplasia congenita, spondylo-costal dysostoses, and diastrophic dysplasia) may have insufficient pulmonary reserves and have sleep-related hypoxemia and hypoventilation. These patients may also have cleft palate and bronchomalacia severe enough to require tracheostomy.

The rarity of these conditions in the general population, combined with the importance of recognizing and managing these specific complications appropriately, were the reasons for convening this expert group. The aim of these best practice guidelines are to assist health care practitioners and sub-specialists in the craniofacial and related management of skeletal dysplasia by: increased awareness of the relevant medical issues; standardizing care pathways; and optimizing outcomes for patients.

## Methods

A RAND-UCLA modified Delphi method was used to create consensus-based guidelines for the treatment and management of craniofacial issues in patients with skeletal dysplasia. This multistep methodology consists of a systematic literature review, creation of a preliminary list of guidance statements, multiple rounds vetting those statements by a group of experts, and a face-to-face meeting where these statements are rated anonymously by the experts.

The panel consisted of 13 international experts on skeletal dysplasia comprising: one pediatric pulmonologist, one pediatric otolaryngologist, six pediatric orthopedic surgeons; four medical geneticists and one pediatric radiologist. The panel has an average of more than 20 years of clinical experience caring for patients with skeletal dysplasia diagnoses. A senior author (MJG) created statements based on a systematic literature review. Two other authors (MBB, DT) further refined the list, generating the first electronic survey consisting of 23 statements. This was distributed with a referenced literature search to a panel of international experts in October 2019 (Round 1). Respondents rated the statements using a 5-point Likert scale (Strongly Agree, Agree, Neutral, Disagree and Disagree Strongly). A month later, at a face-to-face meeting, the results of the first survey and the literature review were presented. Structured discussion was focused on areas of disagreement with the opportunity to modify statements, where required, and further modifications implemented for clarity around specific clinical aspects in reaching consensus. The panel then rated the revised statements anonymously for a second time by electronic survey (Round 2).

Consensus of 80% is considered the standard for agreement in Delphi processes, and this percentage was agreed to a priori. Statements with ≥ 80% of agreement between two rating categories, namely ‘strongly agree’ and ‘agree’, or ‘strongly disagree’ and ‘disagree’, were interpreted to show consensus and therefore included in the guidelines.

## Results

In round one, the panel reached consensus on 13 of 23 statements (Table 1). Statements not reaching 80% agreement are listed in Table 2. During the face-to face meeting, the statements were revised to facilitate reaching consensus and rated again during round two when 22 statements reached ≥ 80% consensus (Table 3); one statement did not (Table 4). These 22 statements are the basis for the best practice guideline presented (Table 5).

**Table 1:** Statements that reached > 80% agreement in Round 1

	Strongly agree	Agree	Neutral	Disagree	Strongly disagree
1. The mortality and morbidity risks for patients with skeletal dysplasia undergoing surgery are greater than the general population	6(50%)	6 (50%)	0	0	0
2. Patients with skeletal dysplasia are more likely than the general population to have abnormal upper airway morphology and function, which can contribute to increased morbidity/mortality	6(50%)	6 (50%)	0	0	0
3. Polysomnography should be considered in the pre-operative assessment of patients with skeletal dysplasia	1 (83%)	9 (75%)	1 (83%)	1 (83%)	0
4. Routine evaluation and surveillance for hearing loss is indicated in patients with skeletal dysplasia	3 (25%)	9 (75%)	0	0	0
5. As children with skeletal dysplasia are at increased risk for hearing loss, audiologic evaluation should be performed on any child with speech delay or a suspicion of hearing difficulties	10(83.3%)	2 (16.7%)	0	0	0
6. Children with skeletal dysplasia and recurrent acute otitis media or with otitis media with effusion of any duration are at increased risk of speech, language, or learning problems	4 (33.3%)	7 (58.3%)	1 (8.3%)	0	0
7. Clinicians may perform tympanostomy tube insertion in children with skeletal dysplasia and unilateral or bilateral otitis media with effusion (OME) that is unlikely to resolve quickly, as reflected by a type B (flat) tympanogram or persistence of effusion for 3 months or longer	1 (8.3%)	9 (75%)	2 (16.7%)	0	0
8. Patients with skeletal dysplasia who have snoring and restless sleep should have polysomnography to diagnose and measure severity of obstructive sleep apnea	7 (58.3%)	5 (41.7%)	0	0	0
9. Children with skeletal dysplasia should undergo polysomnography before tonsillectomy or adenotonsillectomy is performed	4 (33.3%)	6 (50%)	2 (16.7%)	0	0
10. Children with skeletal dysplasia who undergo tonsillectomy and/or adenoidectomy for moderate or severe OSA should be monitored overnight for respiratory difficulties after surgery	4 (33.3%)	8 (66.7%)	0	0	0
11. Children with skeletal dysplasia, especially those with hypognathia or midface hypoplasia have a high risk of malocclusion requiring orthodontic care	2 (16.7%)	8 (66.7%)	2 (16.7%)	0	0
12. Children with skeletal dysplasia should have routine dental care starting in early childhood	6 (50%)	6 (50%)	0	0	0
13. Children with type II collagenopathy have a high risk of hearing loss and palate abnormalities	9 (75%)	1 (8.3%)	2 (16.7%)	0	0

**Table 2:** Statements that did not reach agreement in Round 1

	Strongly agree	Agree	Neutral	Disagree	Strongly disagree
1. As children with skeletal dysplasia are at increased risk of hearing loss, routine hearing screening should be performed at birth, at 12 months of age and annually thereafter	1 (8.3%)	5 (41.7%)	4 (33.3%)	2 (16.7%)	0
2. Children with skeletal dysplasia and acute otitis media should be treated with antibiotics	1 (8.3%)	5 (41.7%)	3 (25%)	3 (25%)	0
3. Children with skeletal dysplasia and otitis media with effusion should be treated with antibiotics	0	3 (25%)	5 (41.7%)	3 (25%)	1 (8.3%)
4. Adenoidectomy and/or tonsillectomy should be the first treatment for documented obstructive sleep apnea in children with skeletal dysplasia	1 (8.3%)	8 (66.7%)	3 (25%)	0	0
5. Children with skeletal dysplasia have a high risk of soft and hard palate abnormalities	1 (8.3%)	6 (50%)	5 (41.7%)	0	0
6. Supernumerary teeth and hypodontia are a frequent accompaniment of certain skeletal dysplasia	4 (33.3%)	5 (41.7%)	2 (16.7%)	1 (8.3%)	0
7. Laryngomalacia and voice abnormalities are a frequent accompaniment s of certain skeletal dysplasia	4 (33.3%)	5 (41.7%)	2 (16.7%)	1 (8.3%)	0
8. In neonates with diastrophic dysplasia and associated SLC26A2 conditions, auricular cystic swelling may progress to permanent deformities. This progression can be lessened by the use of incision and drainage techniques	0	1 (8.3%)	8 (66.7%)	3 (25%)	0
9. In neonates with diastrophic dysplasia and associated SLC26A2 conditions, auricular cystic swelling may progress to permanent deformities. This progression can be lessened by the use of compression molding techniques	0	5 (41.7%)	5 (50%)	1 (8.3%)	0
10. Prior to placement of tympanostomy tubes in children with achondroplasia, clinicians should look for otoscopic signs of a high jugular bulb	5 (41.7%)	4 (33.3%)	3 (25%)	0	0

**Table 3:** Statements that reached > 80% agreement in Round 2, comprising the best practice guidelines

	Strongly agree	Agree	Neutral	Disagree	Strongly disagree
1. Patients with skeletal dysplasia are more likely than the general population to have abnormal upper airway morphology and function, which can contribute to increased morbidity/mortality	13 (100%)	0	0	0	0
2. The mortality and morbidity risks for patients with skeletal dysplasia undergoing surgery are greater than the general population	12 (100%)	0	0	0	0
3. Clinicians should evaluate for signs and symptoms of upper airway obstruction and sleep disordered breathing in patients with skeletal dysplasia at each clinic visit	8 (61.5%)	5 (38.5%)	0	0	0
4. Polysomnography should be performed in patients with skeletal dysplasia who have snoring or signs and symptoms of sleep disordered breathing	9 (69.2%)	4 (30.8)	0	0	0
5. MRI of the craniocervical junction should be considered in infants with achondroplasia and sleep disordered breathing	7 (53.8%)	6 (46.2%)	0	0	0
6. Hearing loss is more prevalent in patients with skeletal dysplasia than in the general population	10(76.9%)	3 (23.1%)	0	0	0
7. Patients with skeletal dysplasia should have hearing assessed at birth or time of diagnosis and at age 5 years	3 (23.1%)	8 (61.5%)	2 (15.4%)	0	0
9. Comprehensive audiologic evaluation should be performed on any child with skeletal dysplasia who has speech delay, suspicion of hearing difficulties, or signs/symptoms of middle ear disease	11 (84.6%)	2 (15.4%)	0	0	0
10.Children with skeletal dysplasia who have otitis media with effusion are at increased risk of speech, language, or learning problems	4 (30.8%)	7 (53.8%)	1 (7.7%)	1 (7.7%)	0
11. Tympanostomy tube insertion may be performed in children with skeletal dysplasia and unilateral or bilateral otitis media with effusion that is unlikely to resolve quickly, as reflected by a type B (flat) tympanogram or persistence of effusion for 3 months or longer	2 (15.4%)	11 (84.6%)	0	0	0
12. At the time of tympanostomy tube placement in children with achondroplasia, the surgeon should look for otoscopic signs of a high jugular bulb	8 (61.5%)	5 (38.5%)	0	0	0
13. Children with skeletal dysplasia and a history of recurrent acute otitis media should be assessed for persistent middle ear disease	5 (38.5%)	8 (61.5%)	0	0	0
14. Children with skeletal dysplasia and acute otitis media should be managed as per established guidelines for the general population	3 (23.1%)	10 (76.9%)	0	0	0
15. Adenoidectomy and/or tonsillectomy should be considered first-line therapy for children with skeletal dysplasia and obstructive sleep apnea	6 (46.1%)	7 (53.9%)	0	0	0
16. Non-invasive positive pressure ventilation is a treatment option for patients with skeletal dysplasia and obstructive sleep apnea	9 (69.2%)	4 (30.8%)	0	0	0
17. Children with skeletal dysplasia should undergo polysomnography before adenoidectomy and/or tonsillectomy is performed	8 (61.5%)	(38.5%)	0	0	0
18. Children with skeletal dysplasia who undergo adenoidectomy and/or tonsillectomy for obstructive sleep apnea should be monitored overnight for respiratory difficulties after surgery	8 (61.5%)	(38.5%)	0	0	0
19. Children with skeletal dysplasia have a higher prevalence of soft and hard palate abnormalities compared to the general population	8 (61.5%)	(38.5%)	0	0	0
20. Children with skeletal dysplasia have a higher prevalence of midfacial, dental and jaw abnormalities compared to the general population	8 (61.5%)	(38.5%)	0	0	0
21. Specialized dental and orthodontic care are part of the core clinical management of patients with skeletal dysplasia, starting in early childhood	9 (69.2%)	4 (30.8%)	0	0	0
22. Stridor or hoarseness in patients who have skeletal dysplasia warrants further evaluation that may include imaging and/or visualization of the larynx	4 (30.8%)	9 (69.2%)	0	0	0
23. In infants with diastrophic dysplasia, auricular cystic swelling may occur. Incision and drainage techniques do not appear to improve outcomes	9 (69.2%)	4 (30.8%)	0	0	0

**Table 4:** Statement that did not reach 80% agreement in Round 2

	Strongly agree	Agree	Neutral	Disagree	Strongly disagree
8. Patients with skeletal dysplasia should have hearing assessed annually until age of 5 years	1 (7.7%)	3 (23.1%)	4 (30.8%)	5 (38.4%)	0

**Table 5:** Actionable recommendations for care of craniofacial aspects of patients with skeletal dysplasia

Recommendation	Action
Clinicians should evaluate for signs and symptoms of upper airway obstruction and for sleep disordered breathing in patients with skeletal dysplasia at each clinic visit	History and clinical exam
Polysomnography should be performed in patients with skeletal dysplasia who have snoring or signs and symptoms of sleep disordered breathing	Polysomnography
MRI* of the cranio-cervical junction should be considered in infants with achondroplasia and sleep disordered breathing	*Magnetic Resonance Imaging
Patients with skeletal dysplasia should have hearing assessed at birth or time of diagnosis and at age 5 years	Audiologic referral
Comprehensive audiologic evaluation should be performed on any child with skeletal dysplasia who has speech delay, suspicion of hearing difficulties, or signs/symptoms of middle ear disease	Audiologic referral
Tympanostomy tube insertion may be performed in children with skeletal dysplasia and unilateral or bilateral otitis media with effusion that is unlikely to resolve quickly, as reflected by a type B (flat) tympanogram or persistence of effusion for 3 months or longer	Otolaryngology referral
At the time of tympanostomy tube placement in children with achondroplasia, the surgeon should look for otoscopic signs of a high and/or dehiscent jugular bulb	Otolaryngology referral
Children with skeletal dysplasia and a history of recurrent acute otitis media should be assessed for persistent middle ear disease	Physical examination and/or Otolaryngology referral
Children with skeletal dysplasia and acute otitis media should be managed as per established guidelines for the general population	Physical examination and/or Otolaryngology referral
Adenoidectomy and/or tonsillectomy should be considered first-line therapy for children with skeletal dysplasia and obstructive sleep apnea	Otolaryngology referral
Children with skeletal dysplasia should undergo polysomnography before adenoidectomy and/or tonsillectomy is performed	Polysomnography
Children with skeletal dysplasia who undergo adenoidectomy and/or tonsillectomy for obstructive sleep apnea should be monitored overnight for respiratory difficulties after surgery	Hospital admission
Specialized dental and orthodontic care are part of the core clinical management of patients with skeletal dysplasia, starting in early childhood	Dental and orthodontic referral
Stridor or hoarseness in patients who have skeletal dysplasia warrants further evaluation that may include imaging and/or evaluation of the larynx	Otolaryngology referral
In infants with diastrophic dysplasia, auricular cystic swelling may occur. Incision and drainage techniques do not appear to improve outcomes	Consider compression moulds

## Discussion

1. Patients with skeletal dysplasia are more likely than the general population to have abnormal upper airway morphology and function, which can contribute to increased morbidity/mortality.

Abnormal airway anatomy and function can be seen in patients with skeletal dysplasia, with specific sites of airway obstruction varying by specific syndromic diagnosis. Craniofacial anomalies, clefts of the palate, structural changes of the upper and lower jaws, and static and dynamic laryngotracheal disease can all contribute to airway obstruction when awake and/or sleeping. For example, those with type II collagen disorders can have tracheomalacia that can further compound anatomically smaller airways [3]. Robin sequence (small jaw with or without cleft palate and relatively large tongue), which is common in certain forms of skeletal dysplasia, can also contribute to upper airway respiratory complications.

2. The mortality and morbidity risks for patients with skeletal dysplasia undergoing surgery are greater than the general population.

The increased mortality and morbidity in patients with skeletal dysplasia who undergo surgery is multifactorial in etiology. Many patients have abnormalities in the development and growth of the craniofacial skeleton leading to restricted opening of the jaw and difficulties in intubation. These risks are compounded by difficult venous access due to joint contractures and/or excess subcutaneous fat, a small thorax, obesity, odontoid hypoplasia with C1-C2 instability, and associated cardiac and other major organ disease [4]. Best practice guidelines for patients undergoing surgery and anesthesia are available in a previous publication from this group [4].

3. Clinicians should evaluate for signs and symptoms of upper airway obstruction and for sleep disordered breathing in patients with skeletal dysplasia at each clinic visit.

Sleep disordered breathing (SDB) comprises a spectrum of conditions including obstructive sleep apnea syndrome (OSAS), central sleep apnea, sleep-related hypoxemia and sleep-related hypoventilation. Patients with skeletal dysplasia are at increased risk for SDB due to the unique craniofacial, tracheobronchial, thoracic cage (i.e. restrictive lung disease) and neurologic features (e.g. hypotonia, respiratory control abnormalities) associated with their specific diagnosis [5, 6]. The signs and symptoms of sleep disordered breathing are non-specific and can be insidious in onset. Disruption of normal sleep architecture and gas exchange abnormalities secondary to SDB lead to neurocognitive problems and negatively impacts cardiovascular function in average stature individuals [7, 8].Although not yet fully investigated in patients with skeletal dysplasia, there is no reason to suspect that these complications would be different. Hence, longitudinal assessment for SDB in *all* skeletal dysplasia patients is required.

Screening [7, 8] should include evaluation for snoring, witnessed apneas, laboured breathing, gasping, snorting, excessive sweating while sleeping, daytime somnolence, behavioral or learning concerns, morning headaches, secondary sleep enuresis and insufficient weight gain in the youngest patients. These questions should be posed at each clinical encounter because they may evolve over time. Certain concomitant findings, such as adenotonsillar hypertrophy or obesity, further increase the likelihood of SDB and strengthen the need for formal evaluation.

To the best of our knowledge, a validated screening tool to screen for sleep disordered breathing in patients with skeletal dysplasia is currently an unmet need.

4. Polysomnography should be performed in patients with skeletal dysplasia who have snoring or signs and symptoms of sleep disordered breathing.

Polysomnography is considered the “gold standard” for the diagnosis of sleep-disordered breathing in all patients. In the clinical setting, reports of excessive snoring and signs and symptoms of SDB in patients with skeletal dysplasia warrant polysomnography. Skeletal dysplasia patients who do not have concerns about their breathing during sleep but have other features of SDB, such as daytime sleepiness, should be considered for polysomnography as the sensitivity and specificity of history and physical exam for the diagnosis of OSAS and sleep -related hypoventilation is poor [7, 9].

Polysomnography is a non-invasive test typically including an electroencephalogram, electro-oculogram, chin and leg electromyogram, electrocardiogram, and pulse oximetry, with assessments of oro-nasal airflow, abdominal and chest wall movements, and video recording. Measurement of partial pressure of carbon dioxide (PCO2) is recommended during standard pediatric polysomnography and is required to diagnose hypoventilation in adults and children. Polysomnography will confirm the presence or absence SDB and provide data on severity, which is useful in short and long-term treatment planning.

Home sleep apnea testing (HSAT) can be utilized to diagnose OSAS in uncomplicated adult patients with increased risk of moderate to severe OSAS and low risk of sleep related hypoventilation [10]. HSAT does not include measurement of PCO2 and therefore cannot be used to diagnose hypoventilation. In adults with a short-statured skeletal dysplasia, the potential for hypoventilation with sleep must be considered, precluding HSAT, but current difficulties with access to adult sleep laboratories and insurance denials for this service may force HSAT to be used as a first-line investigation. HSAT is not recommended for the diagnoses of OSAS in children [11].

Screening modalities for SDB may be appropriate in children when polysomnography is not readily available [7]. These screening options include nocturnal oximetry and daytime nap polysomnography. These screening modalities have weaker positive and negative predicative values when compared to polysomnography, and do not measure partial pressure of carbon dioxide, thereby missing possible hypoventilation. A full polysomnogram is recommended for all “elevated risk” pediatric patients with a skeletal dysplasia, particularly if a screening test was abnormal, signs and symptoms of SDB are reported by the patient or caregiver, and/or if the patient has a skeletal dysplasia significantly affecting the midface, larynx or thorax.

5. MRI of the craniocervical junction should be considered in infants with achondroplasia and sleep disordered breathing.

Compression of the spinal cord at the cervicomedullary level is a recognised complication of achondroplasia in infants, leading to sleep disordered breathing [12]. Whilst there is no clear correlation with severity [13], polysomnographic parameters reveal an association of central apnea with significant cord compression. In this context, obstructive sleep apnoea may also arise, as the consequence of impaired global bulbar muscle tone secondary to neuronal compression at the level of the brainstem [14]. Since this may be amenable to occipital-cervical decompression as opposed to pharyngeal surgery, infants with achondroplasia presenting with significant obstructive sleep apnoea should be investigated using cranial MR imaging.

6. Hearing loss is more prevalent in patients with skeletal dysplasia than in the general population**.**

Most published reports about hearing loss in people with skeletal dysplasia derive from single cases or small cohorts, and often within a single diagnostic group. These reports may also represent an atypical sub-cohort of skeletal dysplasia patients biased towards those seeking medical attention. Nonetheless, it remains the prevalence of hearing deficit is significantly higher than in the general population.

From public health reports and published studies, the prevalence and severity of hearing loss at all ages is well-documented in the general population. Statistics held by the Centers for Disease Control and Prevention (CDC) indicate that 2–3 per 1,000 children in the US have congenital hearing loss in one or both ears [CDC]. With roughly 4,000,000 births per year in the US, this equates to 0.2% born with hearing loss [15]. The prevalence is higher in the age 3–17-years group at 5 in 1,000 children which translates to about 0.5% of this population. [16] Lin et al. [17] concluded from the NHANES dataset that 1 in 8 (13%) US citizens over 12 years of age had hearing loss in one or both ears. A similar prevalence of 14% with hearing loss in adults over 20 years of age was published by Blackwell from The National Health Interview Survey [18]. On a global scale, the WHO estimates at least 5% of the world’s population has hearing loss, which is significant enough to cause major barriers to education, cognitive development and social integration [19].

For comparison, Glass et al. [20] reported a cohort of 17 of 28 (61%) subjects with achondroplasia had hearing loss. Collins [21] reported on serial audiograms of seven subjects with achondroplasia and 100% had hearing loss in one or both ears at one or more visit. Hearing deficit is a well-established component of the natural history of all mucopolysaccharidoses (MPS) marked by chronic middle ear fluid and infection with conductive and sensorineural hearing loss. Though the patient cohorts are small and involve a variety of MPS diagnoses, the majority reported in each study has some type of hearing loss. Bianchi [22] reported hearing loss in 90% of MPS patients affected, 54% of 23 children reported by Vargas-Gamarra [23], and 96% of 53 subjects reported by de Silveira [24]. Skeletal dysplasia in the type II collagen spectrum, such as spondyloepiphyseal dysplasia congenital, Kneist dysplasia and Stickler syndrome, are all characterized by hearing problems. Terhal et al. [25] report 37% of their type II collagen cohort had hearing loss and almost half of these required hearing aids. Hearing loss is also recognized as a common feature of rarer skeletal dysplasia types, including cleidocranial dysplasia [26, 27], the chondrodysplasia punctata group of conditions [28], and Larsen syndrome [29].

In addition to these studies, there is a large survey from a cohort of skeletal dysplasia patients and two population-based studies, all supporting a higher prevalence of hearing loss in skeletal dysplasia when compared to the general population. Hunter et al*.* [30] reported 38% of 193 respondents with a variety of skeletal dysplasia had hearing loss, though audiograms were not available to validate this survey. Machol et al. [31] collected audiometry results from 312 people with osteogenesis imperfecta. They found hearing loss in types I, III and IV combined to be 28%. Tunkel et al*.* [32–34] reported the findings of a hearing screening program conducted in 112 individuals attending a patient support group meeting. Sixty-five percent had achondroplasia and the remainder had one of 11 other skeletal dysplasia conditions. Fifty-two percent of the population were children and of these, 26% failed the hearing screen in one or both ears; of the adults, 55% failed in one or both. Considering other dysplasia diagnoses, 75% with SEDC failed, 66% of those with diastrophic dysplasia, and 66% with Morquio syndrome.

Tympanometry was also assessed in this study and was abnormal in at least one ear in 53% of children participants and 39% of adults. An abnormal tympanometry result was associated with a 9.5 times greater probability of hearing loss in children and 2.8 times greater in the total population.

7. Patients with skeletal dysplasia should have hearing assessed at birth or time of diagnosis and at age 5 years.
8. Patients with skeletal dysplasia should have hearing assessed annually until age of 5 years.

Participants agreed hearing assessment is recommended in patients with skeletal dysplasia, but the need for more frequent (annual) screening did not meet panel consensus level.

There has been tremendous progress in newborn hearing screening for the general population. As of 2017, 98.3% of all infants born in the US were screened for a hearing deficit in the newborn period [35]. Infants and children with skeletal dysplasia should be part of this screened population. Many other developed countries have followed the World Health Organization (WHO) recommendations to establish similar hearing screening programs for all infants and children [36].

However, longitudinal follow-up data from the initial screening is lacking, particularly in the young with underlying medical conditions associated with an increased risk of hearing loss [37]. According to the American Academy of Pediatrics position statement of risk indicators associated with permanent congenital, delayed-onset and/or progressive hearing loss in childhood [38], individuals with skeletal dysplasia meet at least one, and often several, of the identified risk factors. Therefore, the recommendation of Harlor et al*.* [37] is for all children with skeletal dysplasia to have ongoing developmentally appropriate hearing screening and at least one diagnostic audiology assessment by 24 to 30 months of age.

9. Comprehensive audiologic evaluation should be performed on any child with skeletal dysplasia who has speech delay, suspicion of hearing difficulties, or signs/symptoms of middle ear disease.

Comprehensive audiologic assessment differs from hearing screening, with components of testing tailored to the developmental and chronological age of the child. Such testing usually includes pure-tone and speech audiometry and impedance testing (tympanometry and often acoustic reflex testing). The goals of testing are to diagnose hearing loss, obtain ear-specific and frequency specific hearing thresholds and, when possible, distinguish conductive from sensorineural loss. The clinical indicators for suspicion of and referral for evaluation of speech delay or hearing loss are established [37], and these should be applied stringently to children with skeletal dysplasia who frequently have hearing loss with or without Eustachian tube dysfunction. While speech delay can have multiple etiologies, hearing assessment should be part of the evaluation.

10. Children with skeletal dysplasia who have otitis media with effusion are at increased risk of speech, language, or learning problems.
11. Tympanostomy tube insertion may be performed in children with skeletal dysplasia and unilateral or bilateral otitis media with effusion that is unlikely to resolve quickly, as reflected by a type B (flat) tympanogram or persistence of effusion for 3 months or longer.

Children with skeletal dysplasia should be considered “at-risk children” as defined in the AAOHNS *Clinical Practice Guideline: Otitis Media with Effusion (Update)* [39]. This includes those with baseline sensory disturbances, such as visual impairment or permanent hearing loss; developmental delay or pervasive developmental disorders; craniofacial disorders and syndromes that include speech, language, or cognitive issues; suspected or documented speech and language delay; and cleft palate. Such “at-risk children” often have conditions that impair Eustachian tube function with increased frequency and duration of middle ear effusions. Additionally, these children are likely to face a greater impact from the compounding effects of conductive hearing loss resulting from otitis media with effusion. Children in these high-risk groups are usually excluded from longitudinal studies and randomized trials that study the effects of otitis media with effusion on speech and language or the benefits of treatments such as tympanostomy tube placement.

The likelihood of underlying visual impairment, hearing loss, cognitive issues, and cleft palate is dependent upon the underlying type of skeletal dysplasia. One report of a hearing screening program in a mixed population of skeletal dysplasia found that over 25% of children and over 50% of adults failed hearing screening [32, 33] Tympanostomy tubes may be required earlier and more frequently when otitis media with effusion is diagnosed in those with skeletal dysplasia at increased risk. They should also be offered when effusions are long-standing or tympanograms are type B (flat). This is in agreement with the “option” to place tympanostomy tubes for “at-risk” children in a published practice guideline on this surgical procedure [40].

12. At the time of tympanostomy tube placement in children with achondroplasia, the surgeon should look for otoscopic signs of a high and/or dehiscent jugular bulb.

A high or dehiscent jugular bulb in the middle ear is usually asymptomatic, although this anomaly can cause hearing loss or pulsatile tinnitus [41]. The incidence of high jugular bulb with bony dehiscence in the middle ear space appears to be high in achondroplasia, with at least 3.2% of patients in one clinic population having otoscopic or surgical findings consistent with this condition [42]. Severe bleeding can occur when myringotomy is performed. Surgeons should look for otoscopic signs of high jugular bulb prior to myringotomy, and either change position of myringotomy or defer placement of tube(s) when present. Reviews of routine temporal bone CT scans in the general population show high dehiscent jugular bulbs in approximately 3% of cases [43], although clinical experience with myringotomy in children without achondroplasia suggests that clinically relevant jugular anomalies in the middle ear are rare. Jugular bulb abnormalities in achondroplasia may be related to decreased size of the jugular foramen at the skull base as well as enlargement of emissary veins in the skull, as noted in an age-matched controlled study of magnetic resonance imaging [44].

13. Children with skeletal dyslasia and a history of recurrent acute otitis media should be assessed for persistent middle ear disease.
14. Children with skeletal dysplasia and acute otitis media should be managed as per established guidelines for the general population.

While it has been reported that children with achondroplasia frequently experience episodes of acute otitis media [21], it is more likely that these patients (and patients with other skeletal dysplasia diagnoses that have similar craniofacial anatomic issues) have long-term Eustachian tube dysfunction that causes otitis media with effusion, conductive hearing loss, and rarely cholesteatoma [34]. A history of acute otitis media in such patients should be thought of as a signal for further investigation for persistent middle ear disease. The clinician should monitor for clearance of middle ear effusion, and return to normal hearing using audiology testing, with onward otolaryngology referral when needed. The established management algorithms for initial treatment of acute otitis media need no alteration, including decision-making about the need to treat with antibiotics and the choice of antibiotic to be prescribed [45]. There are multiple considerations in deciding initial treatment with antibiotics, including the severity of symptoms, the age of the child, the presence or absence of otorrhea, the involvement of one or both ears, and the preferences of the caregivers [46].

15. Adenoidectomy and/or tonsillectomy should be considered first-line therapy for children with skeletal dysplasia and obstructive sleep apnea.

Adenotonsillectomy is an effective treatment for OSAS in children without coexisting medical issues. First line treatment for OSAS in children with a skeletal dysplasia remains removal of the adenoids and/or tonsils, though with the recognition that this may not be curative [47–49]. Nonetheless, adenotonsillectomy should reduce the number and severity of obstructive events in these patients. A post-operative polysomnography is indicated within a few months after the procedure to confirm if obstruction has been resolved sufficiently to normalize overnight oxygen saturation and CO2 levels [7, 47]. Adenoidectomy alone, versus adenotonsillectomy or tonsillectomy alone, is associated with a higher risk that surgical treatment will fail [47, 50].

16. Non-invasive positive pressure ventilation is a treatment option for patients with skeletal dysplasia and obstructive sleep apnea.

Non-invasive positive pressure ventilation is delivered by nasal mask to open-stent the upper airway and improve lung reserve while sleeping. This is an effective treatment to decrease obstructive events, improve oxygenation and relieve CO2 retention in a patient with OSAS. In children, positive airway pressure (PAP) therapy is recommended if OSAS persists after adenotonsillectomy or if surgery is not pursued [7]. In adult patients, PAP therapy is the first-line treatment for OSAS [51]. Weight loss has also been shown to improve OSAS in patients who are obese and overweight [52, 53].

Other treatment options for OSAS in patients with skeletal dysplasia have been investigated, such as surgical facial skeletal advancement. There are insufficient data supporting the routine use of alternate therapies to treat OSAS in these patients at this time.

17. Children with skeletal dysplasia should undergo polysomnography before adenoidectomy and/or tonsillectomy is performed.

When adenoidectomy and/or tonsillectomy are performed to treat OSAS, children with skeletal dysplasia should undergo polysomnography *before* this surgical procedure to document the presence of OSAS and its severity and *after* surgery to document resolution, especially if the sleep disordered breathing also has a central component. Obtaining a sleep study prior to tonsillectomy for obstructive sleep disordered breathing has been recommended by the American Academy of Otolaryngology-Head and Neck Surgery if patients are less than two years old or have Down syndrome, neuromuscular disorders, sickle cell disease, mucopolysaccharidoses or craniofacial abnormalities [54]. Patients with skeletal dysplasia can be considered as being in the last group, requiring polysomnography to record the presence of OSAS and its severity to plan postoperative and long-term management of the patient. If polysomnography is neither feasible nor readily available and/or if surgery is deemed urgent due to severe signs and symptoms of OSAS, this should be performed as indicated clinically, and not be deferred, to avoid potentially serious patient outcomes, including death.

18. Children with skeletal dysplasia who undergo adenoidectomy and/or tonsillectomy for obstructive sleep apnea should be monitored overnight for respiratory difficulties after surgery.

There is consensus that clinicians should provide overnight inpatient monitoring for high-risk patients undergoing adenotonsillectomy for treatment of obstructive sleep apnea [7, 54]. The possibility of severe respiratory compromise and death in the immediate postoperative period is known to be increased by a number of identified risk factors. Craniofacial anomalies and neuromuscular disorders are included in the group of conditions that should prompt postoperative admission and monitoring [7, 9].

19. Children with skeletal dysplasia have a higher prevalence of soft and hard palate abnormalities compared to the general population.

Cleft palate may occur as an isolated defect or as a component of many syndromes. About 80% of individuals with Robin sequence have an associated syndrome [55]. Similarly, palate abnormalities with or without Robin sequence are more prevalent in certain types of skeletal dysplasia. Specific to skeletal dysplasia, pathogenic variants in genes encoding type II and XI collagen (*COL2A1*, *COL11A1* and *COL11A2*), Filamin-A (*FLNA*), and the diastrophic sulfate transport protein (*SLC26A2*) are all well-known as being associated with cleft palate.

Recognized skeletal phenotypes of type II collagen disorders include (from most to least severe): achondrogenesis type II, hypochondrogenesis, Kniest dysplasia, spondyloepiphyseal dysplasia congenita (SEDC), Stickler syndrome type I, and mild SED with premature arthrosis [3, 56]. Cleft palate is a major feature of SEDC [57–59], but is found in all forms of type II collagen disorders, either alone or as part of Robin sequence. While the majority of people with Stickler syndrome have pathogenic variants in *COL2A1,* approximately 10–20% have mutations in *COL11A1* or *COL11A2*. Furthermore, loss of function mutations in COL11A2 cause otospondylomegaepiphyseal dysplasia (OSMED), a recessively inherited phenotype typically associated with Robin sequence [60] Otopalatal digital (OPD) syndromes types 1 and 2 are the result of mutations in *FLNA*, inherited in an X-linked recessive manner [61]; both are associated with hearing impairment, craniofacial differences including palatal clefts. OPD type II represents the more severe form of the disorder and is typically lethal in infancy [3, 62] Cleft palate abnormalities occur in approximately one-third of patients with diastrophic dysplasia and have been reported in association with Robin sequence in other SLC26A2 disorders, including recessive multiple epiphyseal dysplasia (rMED) [63, 64].

The specialized needs in the treatment of palate abnormalities in the individuals, compounded with the rarity of these conditions, cannot be over emphasized. The necessity to be managed in a centre with expertise in craniofacial and palate surgery and care of patients with skeletal dysplasia is paramount to optimize outcomes [4].

20 Children with skeletal dysplasia have a higher prevalence of mid-facial, dental and jaw abnormalities compared to the general population.

In addition to palatal abnormalities, skeletal dysplasia conditions are frequently associated with midface or maxillary hypoplasia, craniofacial disproportion/asymmetry, and abnormalities of the jaw, such as micrognathia and retrognathia [65]. Craniosynostosis is also associated with several skeletal dysplasias [1], and such patients might benefit from early craniofacial/plastic surgical review for consideration of procedures for functional and/or aesthetic improvement.

Given the associated embryological origins, shared transcription factors and signaling pathways, and common extracellular matrix proteins, disorders of bone formation and modeling are also characterized by dental anomalies [66, 67]. These dental anomalies are therefore not distinct and separate issues, but direct manifestations of the underlying medical condition. Whilst under-modelling of the mandible is characteristic in disorders of type II and XI collagen [68] and diastrophic dysplasia, maxillary hypoplasia is also observed in chondrodysplasia punctata [69] and achondroplasia [70], where the nasal bridge may also be depressed. The nature of the dental anomalies associated with skeletal dysplasia are heterogeneous and can include abnormalities in number, shape, position of the teeth in the jaw, as well as enamel hypoplasia or dentinogenesis imperfecta.

21. Specialized dental and orthodontic care are part of the core clinical management of patients with skeletal dysplasia, starting in early childhood.

The complex dental and craniofacial anomalies seen in skeletal dysplasia conditions present significant challenges in management from an early age. As such, dentists and orthodontists with specialist expertise should be responsible for overseeing surveillance and implementation of appropriate treatment [71]. The objectives are to monitor tooth development and dental health with particular regard to the higher incidence in this population of dental caries, periodontal disease, abscess formation and early dental loss, as well as recording anomalies of the tooth number and morphology, and abnormal dentin and enamel [67]. In this respect, special consideration should be given to cleidocranial dysplasia, where delayed exfoliation of the primary dentition, failure of eruption of the permanent dentition and supernumerary teeth prevail [72], and to osteogenesis imperfecta [73]. Specialist orthodontic treatment is required in conditions related to dental malocclusion, such as achondroplasia [74]. Predisposition to dental abscesses secondary to an elongated dental pulp chamber in hypophosphatemic rickets [75] and early loss of teeth with an intact dental root in hypophosphatasia [67] are two other situations that also warrant special mention in the requirement for expert input. The adverse effects on the teeth and jaw of antiresorptive therapy using bisphosphonates must also be taken into consideration in the management of adults with disorders conferring reduced bone density [76].

22. Stridor or hoarseness in patients who have skeletal dysplasia warrants further evaluation that may include imaging and/or evaluation of the larynx.

Stridor is a sign of upper airway obstruction, and hoarseness is a symptom of abnormality of the larynx specifically at the level of the vocal cords. Upper airway obstruction can be caused by structural changes from the nose and midface to the neck and larynx and down to the chest and trachea. Skeletal dysplasia syndromes can also include upper airway obstruction from disorders of neuromuscular tone associated with any concomitant central nervous system disease. Chronic upper airway obstruction often manifests through changes in voice quality and stridor. [77] Laryngomalacia and tracheomalacia are common in this population [78]. This may worsen with age, particularly in storage disorders such as the mucopolysaccharidoses [79, 80] When stridor and or hoarseness are present, evaluation includes specialty referral as well as evaluation of airway anatomy with endoscopic assessment and/or imaging. This is particularly important prior to surgical procedures and general anesthesia [4].

23. In infants with diastrophic dysplasia, auricular cystic swelling may occur. Incision and drainage techniques do not appear to improve outcomes.

Diastrophic dysplasia is an autosomal recessive dysplasia which affects cartilage and bone development. It is associated with mutations in the SLC26A2 gene. Development of the characteristic enlargement of the external ear, secondary to subchondral cysts, generally occurs in infancy in diastrophic dysplasia. Different interventions have been proposed. Incision and drainage techniques are not recommended and do not lessen the progression. The use of custom compression mouldings may decrease the swelling and help the cosmetic appearancey [81].

## Conclusions

These best practice guidelines were developed to assist health care practitioners and sub-specialists in the craniofacial and related management of skeletal dysplasia. It is hoped that they will increase awareness of the relevant medical issues related to these rare conditions, standardizing care pathways, and improve patient outcomes.

## Data Availability

All data generated or analyzed during this study are included in this published article.
